# Parameter optimization in milling of glass fiber reinforced plastic (GFRP) using DOE-Taguchi method

**DOI:** 10.1186/s40064-016-3055-y

**Published:** 2016-08-22

**Authors:** Sachin Ghalme, Ankush Mankar, Y. J. Bhalerao

**Affiliations:** 1 Department of Mechanical Engineering, SCSM CoE, Ahmednagar, India; 2 Manoharbhai Patel Institute of Engineering & Technology, Gondia, India; 3 VM Institute of Engineering Technology, Nagpur, India; 4 MIT Academy of Engineering, Alandi, Pune, India

**Keywords:** Optimization, GFRP, Design of experiment, Taguchi method

## Abstract

**Introduction:**

Optimization of machining parameters is essential for improving expected outcome of any machining operation.

**Case Description:**

The aim of this work is to find out optimum values of machining parameters to achieve minimal surface roughness during milling operation of GFRP.

**Discussion and Evaluation:**

In this machining operation speed, depth of cut and feed rate are considered as parameters affecting surface roughness and Design of Experiment (DOE)-Taguchi method tool is used to plan experiments and analyse results.

**Conclusion:**

Analysis of experimental results presents optimum values of these three parameters to achieve minimal surface roughness with speed as a major contributing factor. Speed—200 rpm, depth of cut—1.2 mm and feed—40 mm/min are an optimal combination of machining parameter to produce minimal surface roughness during milling of GFRP.

## Background

Glass fiber reinforced plastics (GFRPs) are used in the different field of engineering because of the properties such as high specific strength, high specific stiffness, light weight, high fracture toughness and excellent corrosion and thermal resistance which makes the use of GFRP’s composite especially attractive for aircraft and aerospace application. Because of inhomogeneous nature of the composite material, its response to machining involves various undesirable effects like rapid tool wear, fiber pull-out, surface pitting and delamination. A fiber reinforced composite shows poor surface finish due to fussiness caused by delamination or rupture of fiber. Delamination and cracks in composite finally lead to the poor surface finish. Achieving optimal surface roughness during milling is necessary for the certain application which is affected by various cutting parameters. The knowledge of these parameters would reduce the cost of machining along with improving the quality of work. While milling composite materials, the surface quality (surface roughness) is strongly dependent on machining parameter, tool geometry and cutting forces. Surface roughness is characteristic that could influence the dimensional precision, the performance of mechanical pieces and production cost (Pandurangadu et al. 2010). In this work, an attempt is made towards the optimization of machining parameters for minimization of surface roughness for achieving a desirable quality of milled slots in fiberglass, which in turn critically increase the life of the fiberglass parts. During experimentation, they considered a minimum and maximum range for speed—40 m/min to 200 m/min, depth of cut—0.25 mm to 1.25 mm and feed—0.48 mm/rev to 0.238 mm/rev. Milling of fiberglass is considerably or mostly affected by the tendency of the material itself to delaminate under the action of improper machining parameters. So, it is necessary to select the optimum levels of cutting parameters during the milling operation. Meena Gupta and Surindra Kumar (2015) attempted to investigate the effect of tool nose radius, tool rake angle, feed rate, cutting speed, cutting environment and depth of cut during turning of unidirectional glass fiber reinforced plastics (UD-GFRP) on output response i.e., surface roughness and material removal rate using Taguchi technique. During testing the range of cutting speed, depth of cut and feed rate as 420–1210 rpm, 0.2 mm to 1.4 mm and 0.05 mm/rev to 0.2 mm/rev respectively. Ahmadkhaniha et al. (2015) in their work implemented Taguchi experimental design method to investigate the effect of tool rotational speed, travel speed, tilt angle and penetration depth on the hardness of friction stir welding of Mg. Ashok et al. (2013) in their work predicts the effects of different cutting parameters like tool diameter, feed rate, depth of cut and cutting speed on the quality of the milled slots in the fiberglass plate. In the case of a composite material the machinability is influenced by the type of fiber embedded in the composites. On the other hand, the selection of machining parameters and the machining tool are dependent on the kind of fiber used in the composites and which is crucial in the machining process. Pandurangadu et al. (2010) have attempted to develop a procedure to determine and optimize the selected cutting factors to achieve minimum surface roughness by incorporating response table and response graph, normal probability plot, interaction graphs, and analysis of variance (ANOVA) technique. A machining process involves many process parameters which directly or indirectly influence the surface quality of the product and cost of the product. Glass fiber reinforced polymer (GFRP) composite presents the current trends in the composite technology and machining of these materials is a challenging task (2014). Shunmugesh et al. (2014) proposed L27 orthogonal array for drilling experiments and is used for determining the effect of cutting parameters on surface roughness.

## Design of experiment-Taguchi method

Design of experiment is a powerful technique used for exploring new processes, gaining increased knowledge of the existing processes and optimizing these processes for achieving world-class performance. DOE is implemented in industry for achieving breakthrough improvements in product quality and process efficiency. Application of DOE for researcher helps them to plan their research work and experimentation. The greatest advantage of this method is the saving of experimental time, reducing the cost, and discovering significant factors quickly. Ghalme et al. (2013) implemented DOE-Taguchi technique for evaluating the effect of surface roughness and lubricant viscosity on the coefficient of friction in rolling contact. Considering surface roughness and oil viscosity as two factors, we evaluated the effect on the coefficient of friction, for analysis and experimentation DOE-Taguchi technique implemented successfully. In the case of face milling prediction of cutting parameters as a function of surface roughness, cutting force and temperature is important. Umit Yalcin et al. (2013) presented effect of cutting parameters on mentioned response using ANN using experimental results which were planned using Taguchi method. Nik Mizamzul Mehat and Shahrul Kamaruddin (2011) implemented Taguchi method for optimization of parameters during plastic molding for improving its flexural modulus and flexural strength.

## Experimental procedure

A test specimen of GFRP with dimensions 330 mm × 120 mm × 15 mm thick is used. GFRP is the combination of glass fiber and epoxy resin, which is supplied by Avi Fiber Glass Products & Engineering Works, Ahmednagar. Experiments were conducted on vertical milling machine using cemented carbide end mill (K8), with 8 mm diameter.

### Implementation of Taguchi method

Taguchi method has been used widely in engineering analysis. This technique consists of the planning of experiments with the objective of acquiring data in a controlled way, executing these tests, to obtain information about the behavior of a given process. The experimental work carried out to determine the effect of machining parameters on milling of GFRP composite. To assess the influence of machining parameters on the surface roughness, statistical experiments based on Taguchi design was planned. Considering previous cutting parameters from literature review, during milling the maximum speed selected is only 200 rpm, depth of cut 1.25 mm and feed 0.48 mm/rev (Pandurangadu et al. 2010). So we selected cutting parameters which are not considered in earlier work. Again the available range of cutting parameters with the machine has also considered. The various factors and their levels were chosen for the analysis is presented in Table 1.

**Table 1:** Factors and their levels

Sr. no.	Factor	Levels		
		1	2	3
1	Speed (rpm):N	200	300	550
2	Depth of cut (mm):D	0.6	1.2	1.8
3	Feed (mm/min):F	30	40	100

For three factors at three levels, so the L9 orthogonal array is selected with nine numbers of experiments. Table 2 gives a plan of the experiment.

**Table 2:** L9 orthogonal array

Exp. no.	Control factor		
	N	D	F
1	1	1	2
2	1	2	1
3	1	3	3
4	2	1	3
5	2	2	2
6	2	3	1
7	3	1	1
8	3	2	3
9	3	3	2

### Measurement

The surface roughness of the machined surface was measured using the portable surface roughness tester of Time High Technology Ltd. Make and Model: TR200 with a measurement range of 0.025–12.5 μm. To improve the results, each experiment repeated for four times. Table 3 shows results of the experiment and various values of surface roughness measured for each test.

**Table 3:** Results of experiment

Exp. no.	Surface roughness Ra (μm)				
	1	2	3	4	Avg.
1	1.616	1.894	2.435	2.705	2.1635
2	1.727	1.830	2.235	2.474	2.0665
3	2.926	2.717	2.150	2.898	2.6727
4	2.245	1.691	2.724	3.448	2.5270
5	2.147	2.220	1.924	2.464	2.1887
6	3.152	3.292	2.225	2.568	2.8092
7	2.230	2.167	2.181	2.766	2.3360
8	4.011	2.168	2.453	2.389	2.7552
9	4.439	2.571	2.945	4.291	3.5615

## Analysis of experimental results

### Analysis of signal-to-noise (S/N) ratio

The concept of S/N ratio has been used in the field of acoustics, electrical and mechanical vibrations and other engineering applications. S/N ratio is the ratios of ‘Desired Signal’ representing desired value of output to the ‘noise’ representing the undesirable value, i.e., squared deviation of the output characteristic.

1 $$\text{S}/\text{N} = - 10 \left( {MSD} \right)$$

where, *MSD* = mean squared deviation from the target value of the quality characteristic.

The S/N ratio, which condenses the multiple data points within a trial, depends on the type of characteristic being evaluated. It may be lower is better (LB), nominal is better (NB), higher is better (HB). In this case, the surface roughness is expected to be minimum, so S/N ratio is calculated for lower is better using MINITAB 17 using Eq. 2.

2 $$( S/N )_{LB} = - 10 log\left( {\left( {y_{1}^{2} + y_{2}^{2} + y_{3}^{3} + \cdots +y_{n}^{2} } \right)/n} \right)$$

where *y*_1_, *y*_2_…*y*_*n*_—experimental results/observations and *n*—number of experiments (*y*_1_,…*y*_*n*_).

Table 4 gives S/N ratio for factor and corresponding level.

**Table 4:** S/N ratio (dB)

Level	Speed	DoC	Feed
1	*−7.182*	−7.864	−8.038
2	−7.942	*−7.304*	*−7.437*
3	−6.558	−9.514	−9.208
Delta	2.375	2.211	1.771
Rank	1	2	3

The aim of any experiment is to evaluate the highest possible value of S/N ratio for the result (Roy^1990^). Hence mean squared deviation should be minimum, reflecting a minimum deviation from the target value of the desirable characteristic. Maximization of S/N ratio is significant in any experimentation (Kamaruddin et al. 2010). The high value of S/N ratio signifies the high value of the desired signal than the undesired effect of noise factors. So, during any analysis or experimentation, a high value of S/N ratio is always desirable.

**Fig. 1:**
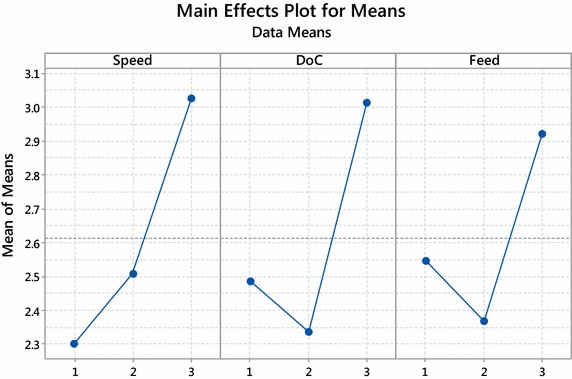
Main effect plot for means

**Fig. 2:**
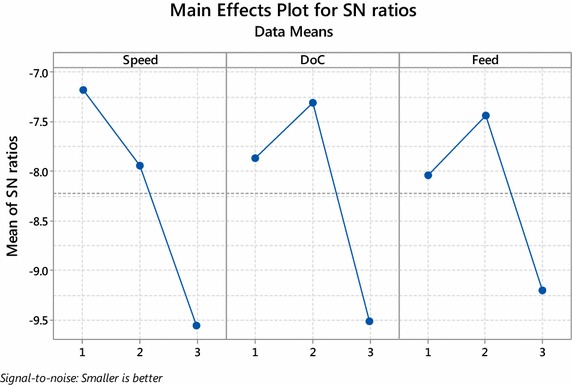
Main effect plot for S/N ratio

Figures 1 and 2 shows Main effect plot for means and Main effect plot for S/N ratio respectively prepared with MINITAB 17. Main effect plot is useful when we have several categorical variables. The main effect is present when the different level of factors affect the desired characteristic differently. It is generated by plotting characteristic average for each factor level. The main effect plot is used to examine the difference between the level means for one or more factors. The main effect plot presents the response mean for each factor level connected by a straight line.

- The lines connecting levels horizontal, indicating there is no main effect and each level of factor affects the characteristic in the same way.
- The lines connecting the levels are not horizontal, indicating there is the presence of the main effect, and each level of factor affects the characteristic differently.

From Figs. 1 and 2 the lines connecting level of factors are not horizontal indicating each factor affects differently. The minimum value of the mean and the maximum value of S/N ratio is observed at level 1 (200 rpm) of speed, level 2 (1.2 mm) depth of cut and level 2 (40 mm/min) feed. These levels of control factor present optimal setting for minimization of surface roughness.

### Analysis of variance (ANOVA)

Experimental results evaluated with software MINITAB 17 for various plots and ANOVA. The ANOVA is performed to investigate the design parameters and to indicate which parameters are significantly affecting the output parameters? This technique does not directly analyze the data, but rather determines the variability (variance) of the data (Roy 1990). Table 5 shows ANOVA table for experimental results calculated at 90 % confidence level.

**Table 5:** Analysis of variance

Source	DF	Seq SS	Contribution (%)	Adj SS	Adj MS	F value
Speed	2	0.84070	50.24	0.84070	0.42035	32.49
DoC	2	0.76115	45.48	0.76115	0.16602	12.83
Feed	2	0.04575	2.73	0.04575	0.02288	1.77
Error	2	0.02588	1.55	0.02588	0.01294	
Total	8	1.67347	100			

In ANOVA table;

- Degrees of freedom (DF) is a measure of amount independent information available from given set of data. DF for concerning factor is one less than the number of levels.
- The sequential or adjusted sum of squares (Seq SS/Adj SS) of factor measures the variability in data contributed by that factor. Total SS is the sum of SS of an individual factor and SS of error.

3 $$Seq\,SS_{factor} = \mathop \sum \nolimits n_{i} \left( {\bar{y}_{i} - \bar{y}} \right)^{2}$$

4 $$Seq\,SS_{error} = \mathop \sum \limits_{i} \mathop \sum \limits_{j} \left( {y_{ij} - \bar{y}_{i} } \right)^{2}$$

5 $$Seq\,SS_{total} = \mathop \sum \limits_{i} \mathop \sum \limits_{j} \left( {y_{ij} - \bar{y}} \right)^{2}$$

where,

$$\bar{y}_{i}-is\,the\,mean\,of\,observations\,at\,i{th}\, factor\,level,$$

$$\bar{y}-mean\,of\,all\,observations, y_{ij}\,{-}\,value\,of\,j{th}\,observation\,at\,the\,i{th}\,factor\,level,$$

$$n_{i}-number\,of\,observations\,for\,the\,i{th}\,factor\,level.$$

- Adjusted mean squares (Adj MS) or variance is Adj SS divided by DF.
- Percentage contribution signifies individual contribution of a factor on the mean response. It is calculated by:
  6 $$\%\,Contribution = \frac{{Seq\,SS_{fator} }}{{Seq\,SS_{total} }}*100$$
- Variance ratio (F value): commonly called as F statistics, is the ratio of variance due to individual factor and variance due to error term.

7 $$Adj\,MS/Variance_{factor} = \frac{{Adj\,SS_{factor} }}{DF}$$

8 $$F\,Value_{factor} = \frac{{Variance_{factor} }}{{Variance_{error} }}$$

From Table 5, it is clear that speed and depth of cut has a significant contribution of 50.24 and 45.48 % respectively on surface roughness during milling of GFRP. While Feed is less significant and contributing only 2.73 % on surface roughness

From F statistic table (Table 6) also it is clear that F value from statistic table for speed and depth of cut is less than F value calculated, signifying that speed and depth of cut have a significant contribution in desired characteristic i.e., surface roughness. While F value from statistic table for feed is more than F value calculated, signifying feed has a less significant effect on desired characteristic i.e., surface roughness.

**Table 6:** F statistics table

Factor	Speed	Depth of cut	Feed
F value calculated	32.49	12.83	1.77
F value from table, F_0.1_(2,2)	9.00	9.00	9.00

## Confirmation experiment

After the optimal level of machining parameters is identified, a confirmation test is necessary to check the accuracy of the analysis. Four experiments conducted with the optimal setting of machining parameters and result presented in Table 7.

**Table 7:** Result of confirmation experiment

Machining parameter	Level	Avg. surface roughness, Ra (μm)
Speed (rpm)	200	2.0225
DoC (mm)	1.2	
Feed (mm/min)	40	

Confirmation experiment presents the minimum value of surface roughness: 2.0225 μm, achieved at optimal machining parameters setting. This value is less than the value obtained for all combinations of parameters during experimentation.

## Conclusion

1. DOE-Taguchi method is very powerful and known technique for analyzing experimental data. Using this technique one can go for optimization of any process. In this paper using DOE-Taguchi method optimization of machining parameters is done for minimization of surface roughness. Results of experiment shows optimum value machining parameters with the corresponding rank:
  - Speed—200 rpm—rank 1.
  - Doc—1.2 mm—rank 2.
  - Feed—40 mm/min—rank 3. These parameters produce a surface roughness of 2.0225 μm.
2. Results of ANOVA presents speed major contributing (50.24 %) factor on surface roughness in the milling of GFRP.
3. It shows the applicability of DOE-Taguchi method in production planning and analysis for improving process outcome.
